# Indicators for early assessment of palliative care in lung cancer patients: a population study using linked health data

**DOI:** 10.1186/s12904-018-0285-5

**Published:** 2018-02-26

**Authors:** Maria Kelly, Katie M. O’Brien, Michael Lucey, Kerri Clough-Gorr, Ailish Hannigan

**Affiliations:** 1 National Cancer Registry Ireland and Graduate Entry Medical School, University of Limerick, Limerick, Ireland; 2 National Cancer Registry Ireland and Cork Institute of Technology, Cork, Ireland; 3 Milford Care Centre, Castletroy, Limerick, Ireland; 4 Graduate Entry Medical School, University of Limerick, Limerick, Ireland

**Keywords:** Lung cancer, Palliative care, Survival time, Administrative health data

## Abstract

**Background:**

Analysing linked, routinely collected data may be useful to identify characteristics of patients with suspected lung cancer who could benefit from early assessment for palliative care. The aim of this study was to compare characteristics of newly diagnosed lung cancer patients dying within 30 days of diagnosis (short term survivors) with those surviving more than 30 days. To identify indicators for early palliative care assessment we distinguished between characteristics available at diagnosis (age, gender, smoking status, marital status, comorbid disease, admission type, tumour stage and histology) from those available post diagnosis. A second aim was to examine the association between receiving any tumour-directed treatment, place of death and survival time.

**Methods:**

A retrospective observational population based study comparing lung cancer patients who died within 30 days of diagnosis (short term survivors) with those who survived longer using Chi-squared tests and logistic regression. Incident lung cancer (ICD-03:C34) patients diagnosed 2005–2012 inclusive who died before 01–01-2014 (*n* = 14,228) were identified from the National Cancer Registry of Ireland linked to death certificate data and acute hospital episode data.

**Results:**

One in five newly diagnosed lung cancer patients died within 30 days of diagnosis. After adjusting for stage and histology, death within 30 days was higher in patients who were aged 80 years or older (adjusted OR 2.46; 95%CI 2.05–3.96; *p* < 0.001), patients with emergency admissions at diagnosis (adjusted OR 2.96; 95%CI 2.61–3.37; *p* < 0.001) and patients with any comorbidities at diagnosis (adjusted OR 1.32 95%CI 1.15–1.52; *p* < 0.001). Overall, 75% of those who died within 30 days died in hospital compared to 43% of longer term survivors.

**Conclusions:**

We have shown a high proportion of lung cancer patients who die within 30 days of diagnosis are older, have comorbidities and are admitted through the emergency department. These characteristics, available at diagnosis, may be useful prognostic factors to guide decisions on early assessment for palliative care for lung cancer patients. Patients who die shortly after diagnosis are more likely to die in hospital so reporting place of death by survival time may be useful to evaluate interventions to reduce deaths in acute hospitals.

## Background

Worldwide, lung cancer remains the most common cancer and was the cause of over 1.6 million deaths in 2012. In Europe 12.1% of all incident cancers in 2012 were lung cancer and it accounted for 20.1% of all cancer deaths [1]. Typically lung cancer is characterised by short survival times, often attributed to late diagnosis [2, 3]. The UK and Ireland have the poorest one- and five- year relative survival rates for lung cancer in Northern Europe [4]. In the UK just over one third of patients survive more than 1 year following a lung cancer diagnosis (2010–2011) [5].

Most patients with advanced cancer die in hospital [6] although a review of 210 studies from 33 countries found most people would prefer to die at home [7]. Bekelman et al. reported death rates in acute hospitals ranging from 54% in Canada to just over 20% in the United Sates for lung cancer decedents over 65 years of age in 2010 across 6 developed countries [8]. Early palliative care in patients with metastatic non-small-cell lung cancer has been found to improve survival times and lead to less aggressive care at end of life compared to patients receiving standard care [9]. It also improves patients understanding of their prognosis which may lead to more informed choices near end of life [10].

Identifying those in need of early assessment for palliative care involves understanding prognostic factors of survival i.e. factors measured before treatment that have an impact on a patient’s outcome “independently” of received or general class of treatment (Paesmans, 2012). In the case of lung cancer clinical stage and functional status are two important prognostic factors for survival while age and gender are, to a lesser extent, also important [11]. The potential of using linked routine health data for prognostication has increasingly been recognised [12]. Data from multiple sources (registries, death certificates, hospital and community-based healthcare records) can be used retrospectively to identify those with unique needs or at risk of poorer outcomes [13–16].

The aim of this study was to use linked routine data to compare characteristics of newly diagnosed lung cancer patients dying within 30 days of diagnosis (short term survivors] with those surviving more than 30 days. To identify indicators for early palliative care assessment we distinguished between characteristics available at diagnosis (age, gender, smoking status, marital status, comorbid disease, admission type, tumour stage and histology) from those available post diagnosis. A second aim was to examine the association between receiving any tumour-directed treatment, place of death and survival time.

## Methods

### Data sources

The data sources for this study were the National Cancer Registry Ireland (NCRI), the hospital inpatient enquiry (HIPE) database from the Economic and Social Research Institute (ESRI) and death certificate (DC) data from the Central Statistics Office (CSO).

The NCRI records demographic, clinical and treatment information for all cancers diagnosed in the Irish population, according to internationally accepted conventions. The registry obtains data on patients with cancer from a variety of sources but primarily via qualified cancer data registrars who are employed by the registry and based in the major acute hospitals around the country. The main source of notification of each new case is a pathology report. The second main source of notification is HIPE. HIPE is an electronic based information system that records demographic, clinical and administrative data on discharges from all acute public hospitals in Ireland. Each public hospital produces an annual HIPE list which identifies all cases of cancer discharged from the hospital during that year; HIPE allows the registry to identify cases that have not had a histological verification of the diagnosis, or for which the registry failed to identify a pathology report. For data confidentiality reasons, only HIPE inpatient episodes that mention a cancer diagnosis are made available to the registry so episodes prior to a cancer diagnosis are not provided. It is a legal requirement in Ireland that every death that takes place in the State must be recorded and registered within 3 months. Population based death certificate data on date, cause and place of death of all decedents is provided by the CSO.

The cancer registry routinely links HIPE data and death certificate data to cancer registry data using probabilistic matching techniques, for case ascertainment and verification purposes. Completeness of case ascertainment for lung cancer is estimated to be 98.7% [17].

### Data definitions

Information collected by the registry is coded and classified according to international guidelines including international classification of diseases (ICD) codes, definitions of incidence and how multiple tumours are handled [18]. The date of diagnosis is taken as the date of incidence which is selected from a hierarchy of dates in the following order, the date of first histological confirmation of malignancy or in the absence of histological confirmation, the date of first treatment (excluding “seen but not treated”), followed by the date of admission to hospital because of the malignancy [18, 19]. Stage at diagnosis was defined according to American Joint Committee on Cancer (AJCC) summary staging [20]. Histological groupings were based on the International Agency for Research on Cancer classification [21]. Treatment data were classified to a yes/no category for any tumour-directed surgery, chemotherapy or radiotherapy received within 1 year of diagnosis.

The diagnosis episode was the inpatient episode during which the lung cancer diagnosis was made. For a small proportion of patients (10%) the diagnosis date didn’t occur during an inpatient episode so the episode occurring in the interval from 7 days before to 14 days after the lung cancer diagnosis date was used. HIPE records episode admissions as either emergency or elective. Emergency admissions occur when a patient requires immediate care and treatment as a result of a severe, life threatening or potentially disabling condition with the patient generally admitted through the Emergency Department. Elective admissions occur when the patient’s condition permits adequate time to schedule the availability of suitable services to the patient [22]. A co-morbidity score for each patient, based on the updated Charlson index [23] was derived from all diagnoses recorded in HIPE for the diagnosis episode. The Charlson comorbidity index (CCI) predicts the one-year mortality for a patient who may have a range of 17 comorbid conditions. Each condition is assigned a score of 1, 2, 3, or 6, depending on the risk of dying associated with each one. Scores are summed to provide a total score to predict mortality. Patients were classified to three comorbidity categories ‘None’, ‘1’ and ‘> 1’ based on their Charlson score. The lung cancer diagnosis was disregarded when calculating the co-morbidity score. For every episode, HIPE records a discharge code describing where the patient was discharged to, including categories for home, nursing home, died with and without post mortem and transfer to another hospital. For this analysis we classified discharge code to ‘Death’ and ‘Other’.

### Setting/participants

All incident lung cancer patients (ICD-O3:C34) [24], who were diagnosed in Ireland between 2005 and 2012 and who died before 01–01-2014 were identified from the NCRI. These records were linked to HIPE and death certificate data.

### Outcome variable

To identify early indicators for palliative care assessment, patients were classified to those who died within 30 days of diagnosis (short term survivors) and those who survived more than 30 days. We chose the 30 day cut-off for a number of reasons i.) 30 day mortality has been used previously as an indicator of early mortality for newly diagnosed breast or colorectal cancer patients in Scotland [25] and to assess factors affecting 30-day mortality in a national patient population of lung and breast cancer patients receiving systemic anti-cancer therapy [26], ii.) it aligns with our study objective of identifying patient cohorts who might benefit from early assessment for palliative care as these patients are unlikely to have gained survival benefits of treatment, iii.) lung cancer is characterised by short survival times and in our study just over 20% of patients died within 30 days of diagnosis providing a natural cut point for this analysis and iv.) it is easily derived from routine data and can facilitate comparisons across health systems.

The association between receiving any tumour-directed treatment, place of death and survival time as a continuous variable was explored graphically.

### Statistical analysis

Chi-squared tests for independence were used to test for significant associations between categorical variables i.e. patient demographic variables present at diagnosis (age, gender, smoking status and marital status); clinical variables at diagnosis (stage, histology and any comorbidities); characteristics of the diagnosis episode (admission type i.e. elective or emergency and whether death occurred in hospital during the diagnosis episode); post diagnostic characteristics including receipt of any tumour-directed treatment, cause of death and place of death; and survival time (≤ 30 days, > 30 days) using a 5% level of significance. Cramer’s V was used as a measure of the strength of the association with nominal variables. Somers’ D was used as a measure of the strength of the association between ordinal independent variables and stratified survival time.

Multivariable logistic regression analysis was used to predict death within 30 days of diagnosis (yes, no). The model was fitted in a two stage process; the first stage examined the impact of patient characteristics immediately available at presentation. Next, the impact of clinical variables (e.g. histology and staging of tumour) were assessed by adding these to the model. Model goodness-of-fit was checked using the Hosmer and Lemeshow test [27]. The R statistical package was used for data analyses [28].

## Results

Of the 16,638 incident lung cancer patients diagnosed between 2005 and 2012 inclusive, 14,228 (85.5%) died before 01–01-2014. Of these, 383 cases were notified from death certificate data only and due to insufficient data are excluded from the analysis, leaving 13,845 cases in the final dataset. Median survival time was 137 days with an interquartile range (IQR) of 44–339 days.

Almost one in five (*n* = 2595, 18.7%) newly diagnosed lung cancer patients died within 30 days of diagnosis. Table 1 shows the characteristics of the short term survivors compared to those who lived more than 30 days. The strongest association with short term survival were emergency admission at diagnosis, comorbidities at diagnosis, tumour histology and tumour stage. Compared with longer-term survivors, short term survivors were more likely to be admitted as emergencies at diagnosis (84% versus 52%), they were more likely to have comorbidities at diagnosis (18% versus 13% had a Charlson score of 1 and 19% versus 12% scored > 1), and were less likely to have their tumour characterised (37% versus 15% were histologically unspecified and 18% versus 11% were unstaged). Table 2 shows the results of the multivariable logistic regression analysis adjusted for characteristics available at the diagnosis episode. These include gender, marital status and comorbidities present at diagnosis. Older patients (OR 2.72; 95%CI 2.29–3.24; *p* < 0.001), those with emergency admissions at diagnosis (OR 3.92; 95%CI 3.47–4.44; *p* < 0.001) and those with comorbid disease at diagnosis were significantly more likely to die within 30 days of diagnosis; the odds increased from 1.23 (95%CI 1.08–1.41; *p* = 0.002) among patients with a CCI score of 1 to 1.41 (95%CI 1.23–1.62; *p* < 0.001) among patients with a CCI > 1. Table 3 shows the results of the regression analysis adjusted for the clinical variables stage and histology. After adjusting for stage and histological type, older age (adjusted OR 2.46; 95%CI 2.05–2.96; *p* < 0.001) emergency admission at diagnosis (adjusted OR 2.96; 95%CI 2.61–3.37; *p* < 0.001) and comorbid disease were still strongly associated with death within 30 days of diagnosis; the adjusted odds increased from 1.32 (95%CI 1.15–1.52; *p* < 0.001) among patients with a CCI score of 1 to 1.44 (95%CI 1.25–1.65; *p* < 0.001) among patients with a CCI > 1.

**Table 1:** Patient, tumour and admission characteristics at diagnosis, by survival category

	Survival category			
	0–30 days *n* = 2595	> 30 days *n* = 11,250	All *n* = 13,845	Tests of association
Age group				
< 60	253 (10)	1990 (18)	2243 (16)	χ^2^ = 268.78, df(3), *p* < 0.001 Somers’ d = 0.082
60–69	569 (22)	3240 (29)	3809 (28)	
70–79	950 (37)	3835 (34)	4785 (35)	
80+	823 (32)	2185 (19)	3008 (22)	
Gender				
Male	1606 (62)	6671 (59)	8277 (60)	χ^2^ = 5.88, df(1), *p* = 0.015 Cramer’s V = 0.021
Female	989 (38)	4579 (41)	5568 (40)	
Marital status				
Partner	1224 (47)	6193 (55)	7417 (54)	χ^2^ = 52.66, df(1), *p* < 0.001 Cramer’s V = 0.062
Other	1371 (53)	5057 (45)	6428 (46)	
Smoker				
Ever	1803 (69)	8641 (77)	10,444 (75)	χ^2^ = 78.77, df(3), *p* < 0.001 Cramer’s V = 0.075
Never	219 (8)	915 (8)	1134 (8)	
Unknown	573 (22)	1694 (15)	2267 (16)	
Diagnosis episode admission^a^				
Elective	360 (16)	3990 (46)	4350 (40)	χ^2^ = 635.33, df(1), *p* < 0.001 Cramer’s V = 0.242
Emergency	1823 (84)	4669 (54)	6492 (60)	
Diagnosis episode Charlson score^a^				
0	1362 (62)	6481 (75)	7843 (72)	χ^2^ = 134.08, df(2), *p* < 0.001 Cramer’s V = 0.114
1	403 (18)	1166 (13)	1569 (15)	
> 1	418 (19)	1012 (12)	1430 (13)	
Stage^b^				
Stage 0/I/II	183 (7)	2148 (19)	2331 (17)	χ^2^ = 365.11, df(3), p < 0.001 Somers’ d = 0.098
Stage III	573 (22)	3246 (29)	3819 (28)	
Stage IV	1376 (53)	4606 (41)	5982 (43)	
Un-staged	463 (18)	1250 (11)	1713 (12)	
Histology^c^				
Adenocarcinoma	447 (17)	3027 (27)	3474 (25)	χ^2^ = 735.46, df(5), *p* < 0.001 Cramer’s V = 0.23
Carcinoma	254 (10)	1346 (12)	1600 (12)	
Large cell carcinoma	121 (5)	421 (4)	542 (4)	
Unspecified malignant	951 (37)	1715 (15)	2666 (19)	
Small cell carcinoma	449 (17)	1708 (15)	2157 (16)	
Squamous cell carcinoma	373 (14)	3033 (27)	3406 (25)	

^a^ The diagnosis episode was the episode where the lung cancer diagnosis was made or the first episode occurring in the interval from 7 days before to 14 days after the lung cancer diagnosis. Episodes outside this interval were excluded from the analysis, *n* = 1591. There was no matching HIPE data for *n* = 1412 decedents. The denominator data is *n* = 2183 for short term survivors and *n* = 8659 for longer term survivors

^b^ Due to small cell numbers (*n* = 11), ‘Stage 0’ classification was merged with ‘Stage I/II’ classification

^c^ Due to small cell numbers (*n* = 21), ‘Sarcoma’ classification was merged with ‘unspecified malignant’ classification. Morphologies based on International Agency for Research on Cancer classification [21]

**Table 2:** Characteristics available at diagnosis associated with death within 30 days of diagnosis

Characteristic	Adjusted odds ratio	95% confidence interval		*p*-value for Wald test
Age group				
< 60 years	1.00			
60–69 years	1.44	1.21	1.72	< 0.001
70–79 years	1.91	1.62	2.25	< 0.001
80+ years	2.70	2.27	3.22	< 0.001
Marital status				
Partnered	1.00			
Other	1.19	1.07	1.31	0.001
Gender				
Male	1.00			
Female	0.81	0.73	0.90	< 0.001
Smoker				
Ever	1.00			
Never	0.98	0.82	1.17	0.906
Unknown	1.48	1.29	1.68	< 0.001
Diagnosis episode admission^a^				
Elective	1.00			
Emergency	3.92	3.47	4.44	< 0.001
Diagnosis episode Charlson score^a^				
0	1.00			
1	1.23	1.08	1.41	0.002
> 1	1.41	1.23	1.62	< 0.001

^a^ The diagnosis episode was the episode where the lung cancer diagnosis was made or first episode occurring in the interval of 7 days before to 14 days after the lung cancer diagnosis

**Table 3:** Characteristics and clinical data available at diagnosis associated with death within 30 days of diagnosis

Characteristic	Adjusted odds ratio	95% confidence interval		p-value for Wald test
Age group				
< 60 years	1.00			
60–69 years	1.45	1.22	1.74	< 0.001
70–79 years	1.99	1.68	2.36	< 0.001
80+ years	2.44	2.03	2.94	< 0.001
Marital status				
Partnered	1.00			
Other	1.15	1.04	1.28	0.007
Gender				
Male	1.00			
Female	0.77	0.69	0.86	< 0.001
Smoker				
Ever	1.00			
Never	0.91	0.75	1.09	0.313
Unknown	1.27	1.10	1.46	< 0.001
Diagnosis episode admission^a^				
Elective	1.00			
Emergency	2.96	2.61	3.37	< 0.001
Diagnosis episode Charlson score				
0	1.00			
1	1.32	1.15	1.52	< 0.001
> 1	1.44	1.25	1.65	< 0.001
Stage				
Stage 0/I/II	1.00			
Stage III	2.12	1.73	2.62	< 0.001
Stage IV	3.37	2.77	4.11	< 0.001
Un-staged	3.47	2.76	4.37	< 0.001
Histology				
Adenocarcinoma	1.00			
Carcinoma	1.17	0.97	1.41	0.095
Large cell	1.71	1.32	2.20	< 0.001
Unspecified malignant	2.91	2.49	3.41	< 0.001
Small cell	1.50	1.28	1.76	< 0.001
Squamous cell	0.84	0.71	0.98	0.034

^a^ The diagnosis episode was the episode where the lung cancer diagnosis was made or first episode occurring in the interval of 7 days before to 14 days after the lung cancer diagnosis

**Fig. 1:**
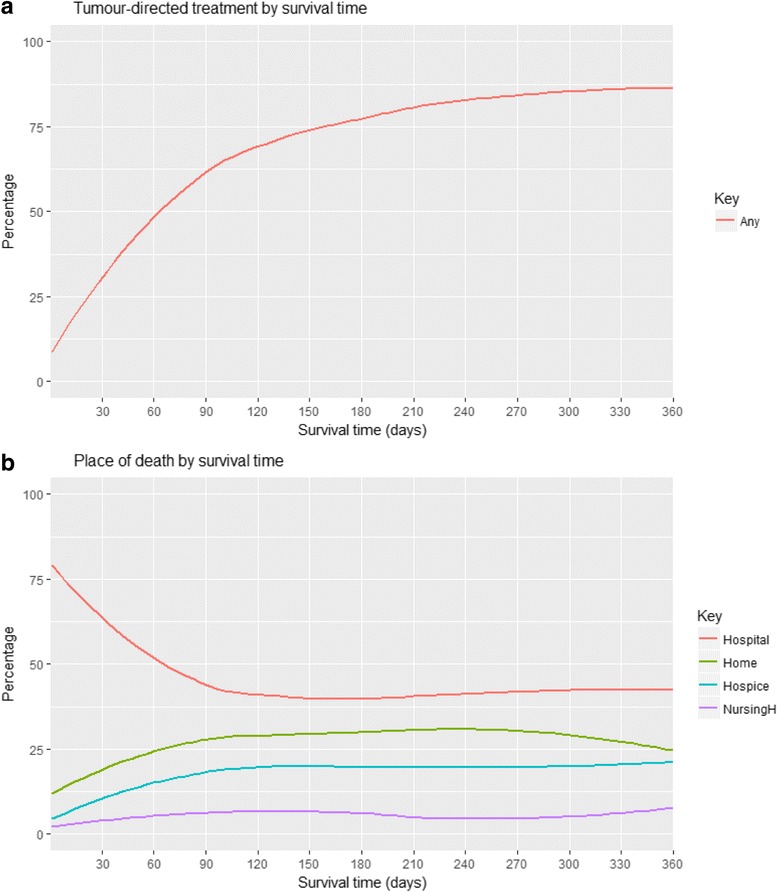
Percentage (**a**) receiving tumour–directed treatment and (**b**) place of death by survival time

Table 4 describes treatment received post diagnosis, the discharge code for the diagnosis episode, the place of death and the cause of death by survival category. Only 16% of the short term survivors received tumour directed treatment post diagnosis compared to 72% of the longer term survivors. Increasing survival time was associated with a steady increase in the percentage receiving treatment (Fig. 1a).

**Table 4:** Post diagnosis characteristics; treatment, place of death and cause of death

	Survival category			
	0–30 days *n* = 2595	> 30 days *n* = 11,250	All *n* = 13,845	Tests of association
Tumour directed treatment^a^				
None	2169 (84)	3183 (28)	5352 (39)	χ^2^ = 2718.36, df(1), *p* < 0.001, Cramer’s V = 0.443
Any	426 (16)	8067 (72)	8493 (61)	
Diagnosis episode discharge code^b^				
Death	1243 (57)	237 (3)	1480 (14)	χ^2^ = 4345.6 df(1), *p* < 0.001, Cramer’s V = 0.633
Other	940 (43)	8422 (97)	9362 (86)	
Place of death				
Hospital	1949 (75)	4850 (43)	6799 (49)	χ^2^ = 876.52, df(5), *p* < 0.001, Cramer’s V = 0.252
Home	338 (13)	3200 (28)	3538 (26)	
Hospice	185 (7)	2084 (19)	2269 (16)	
Nursing Home	65 (3)	688 (6)	753 (5)	
Unknown	34 (1)	339 (3)	373 (3)	
No death certificate	24 (1)	89 (1)	113 (1)	
Cause of death				
Lung cancer	2320 (89)	10,081 (90)	12,401 (90)	χ^2^ = 0.096, df(1), *p* = 0.757, Cramer’s V = 0.003
Other	275 (11)	1169 (10)	1444 (10)	

^a^ Refers to tumour directed treatment received within 1 year of diagnosis

^b^ The diagnosis episode was the episode where the lung cancer diagnosis was made or the first episode occurring in the interval from 7 days before to 14 days after the lung cancer diagnosis. Episodes outside this interval were excluded from the analysis, *n* = 1591. There was no matching HIPE data for *n* = 1412 decedents. The denominator data is *n* = 2183 for short term survivors and *n* = 8659 for longer term survivors

The proportion dying in hospital decreased as survival time increased to 90 days but tended to remain stable after 90 days survival (Fig. 1b). Overall 75% of the short term survivors died in hospital compared to 43% of longer term survivors. The proportion recorded as dying from lung cancer was similar in both groups - 89% short term survivors and 90% longer term survivors had lung cancer recorded as main cause of death.

## Discussion

One in five newly diagnosed lung cancer patients in our study died within 30 days of diagnosis and one quarter died within 44 days. Given the very short survival times, indicators for early assessment for palliative care are important to facilitate ongoing management and end of life care. We identified that older patients (aged 80 years and over), with any comorbid disease, who have emergency admissions at diagnosis are more likely to die within 30 days following diagnosis than younger patients, without comorbidities and admitted electively.

### Survival time

Our study aimed to identify indicators for early assessment for palliative care using routine linked data to compare characteristics of short- and longer term survivors. Survival time is affected by many factors including patient age, functional status, tumour stage at diagnosis and the treatment modalities available to treat disease. The relationships between these factors are complex; treatment plans are optimised to the individual and patients well enough to receive curative treatment will derive a survival benefit from that treatment. Patient who die shortly after diagnosis may have had too little time for adequate assessment and appropriate care plans (curative or palliative) to be put in place however, this group has not been well characterised at the population level. We are aware of only one population based study characterising short term survivors [25]. We have shown patients surviving longer are more likely to receive tumour-directed treatment which is associated with survival benefit, the proportion of patients receiving treatment increased steadily with increasing survival time.

Retrospective studies of cancer care often use look-back periods from death of 6 or 12 months [29]. Survival times vary considerably by cancer type and stratification by survival time from diagnosis could be more informative than look-back studies (using defined periods of time before death). For example when examining the care received at end of life, aggressive care may be completely appropriate in the early stages of treatment shortly after a cancer diagnosis however this information can be lost in look-back studies if survival time is not reported. Reporting survival time provides an added context to evaluate the care patients receive at end of life. As we have shown it can highlight opportunities to improve that care for patient subgroups, for example patients who die very soon after diagnosis.

### Indicators for palliative care

#### Age and emergency admission

In a critical review of 10 studies from the USA, Canada, France, Australia and New Zealand, Wong et al. highlighted the absence of evidence regarding the palliative care requirements of older patients particularly those presenting to emergency departments, with a call for more research to help improve service provision [30]. Similarly Brewster et al. [25] found patients dying within 30 days of diagnosis were more likely to be elderly and have one or more emergency admission in the 30 days either before or after diagnosis. Our population based study supports the international evidence for high levels of emergency admissions and poorer survival in older cancer patients.

Sixty percent of patients diagnosed with lung cancer in our study presented as an emergency admission. McPhail et al. also found emergency presentation remains predictive of short-term mortality in cancer patients even when age, stage, and co-morbidity are accounted for [31]. A prospective mixed methods single centre study of lung cancer patients presenting as emergencies reported palliative care needs were high and various information and support needs unmet [32]; the authors recommended a specialist palliative care assessment be routinely offered.

A lack of access to cancer diagnostics is a recognised short-coming in the Irish health system which has led to increased referrals of patients to emergency departments by general practitioners [33] and to initiatives to improve access to care. In 2012, the National Cancer Control Programme in Ireland initiated rapid access clinics providing direct access to consultant led assessment and diagnostic services for patients with suspected lung disease or cancer [34]. The clinics allow for suspect cases to be fast tracked and diagnosed on an urgent basis thereby facilitating earlier diagnosis and increased survival. Our study results (on patients diagnosed from 2005 to 2012) provides baseline data on stage at diagnosis and survival for patients diagnosed before the introduction of these rapid access clinics.

#### Comorbidity

Comorbid disease has been shown to delay diagnosis in colorectal cancer patients and particularly in older patients [35]; comorbid conditions were classified as ‘competing demands’ (unrelated to colorectal cancer) or ‘alternative explanations’ (sharing symptoms with colorectal cancer). In a prospective study across five US hospitals, earlier consultation with specialist palliative care teams was associated with lower cost of hospital stay for patients admitted with an advanced cancer diagnosis [36]. A second related study showed the effect was larger for patients with higher number of comorbidities [37]. Comorbidity was measured using the Elixhauser comorbidity index [38] which counts the presence of thirty one serious conditions. We used the Charlson index [23] to derive a comorbidity score from the diagnosis episode as we did not have access to HIPE data before the cancer diagnosis. Furthermore under recording of comorbid disease in the HIPE data is a potential limitation and as other measures such as functional status were not available to us, it is probable we have under estimated the level of comorbid disease in our patient sample. Notwithstanding, we have shown short term survivors have more comorbid disease than longer term survivors and the odds of early mortality increases with increased comorbidity. Given this, earlier referral for palliative care assessment could not only improve the patient experience at end of life but might also yield economic benefits.

### Stage

Our study also reports short term survivors were more likely to have tumours which are less well characterised, (i.e. not staged and histologically unspecified), than patients who survive longer. Current national guidelines recommend patients with stage IV non-small-cell lung cancer should be offered concurrent specialist palliative care and standard oncological care at initial diagnosis [39] (see recommendation 2.8.1.1 page 109). It further recommends all patients with advanced stage lung cancer should have their palliative care needs assessed. Using the stage IV criteria as an indicator for palliative care just over 50% of the short-term survivors from our study would be identified and this highlights the need for additional prognostic indicators for early assessment.

#### Place of death

Our rate of death in acute hospitals of 49% for lung cancer decedents is higher than that reported by Bekelman et al. for lung cancer decedents aged 65 years or older in acute hospitals in England (42.6%), Germany (45%), The Netherlands (29.5%), Norway (46.5%) and the United States (20.2)% and lower than Canada (54.1%) [8]. The higher percentage of hospital deaths in our study is partially due to the high rates in the short term survivors, but also the Bekelman study was restricted to deaths for 1 year (2010) while our study had a broader time frame of 8 years and included all lung cancer patients. Moves to reduce deaths in acute hospitals adopted in the United States and the Netherlands (as described by Bekelman et al.) have been effective however without reporting place of death by survival time, it is unclear whether they benefit short term survivors.

In an economic evaluation of specialist palliative care services in three parts of Ireland which have heterogeneous structures and resources for these services, Brick et al. found that an area with well-developed specialist palliative care services and, where its role is understood, is likely to have more referrals and that these will in general be earlier [40]. O’Leary et al., in a study of one specialist palliative care service in Ireland over a 6-month period found late referral to palliative care was associated with receiving specialist palliative care in one care setting only but receiving care across multiple settings supported people to stay at home for longer [41].

Death within 30 days of diagnosis means there is little time to determine and put appropriate care plans in place and death in hospital for these patients might be entirely appropriate. In this context triggers for early assessment of palliative care for newly diagnosed lung cancer patients are very important so that the best care can be provided as soon as possible whether in hospital, in the community or at home.

### Strengths and limitations

A strength of our study is the use of high quality population based cancer registry data which has been verified and augmented by linkage to hospital episode data to death and certificate data. Linked datasets provide novel opportunities for research at the population level, however there are limitations to their use. For data confidentiality reasons, we cannot access hospital episode data for patients before a cancer diagnosis, so cannot examine health care utilisation leading to diagnosis. This information would facilitate a more accurate profile of the short term survivors in particular; multiple morbidities, especially in elderly patients, might explain short survival and/or post-mortem diagnoses of lung cancer. In this study 10% of lung cancer cases recorded by NCRI had no corresponding HIPE record. Failure to find a match can occur for several reasons including: typographical errors in fields used for matching, missing data on either system or no mention of cancer on the HIPE record, in which case the record would not be made available to NCRI. A cross reference of HIPE data with death certificate data indicate 5% of patients with place of death recorded as hospital do not have a HIPE record and we estimated 3% of these died in private hospitals who do not provide HIPE data.

## Conclusion

A major focus of end-of-life care research has been to identify cohorts of patients who may be near end of life and would benefit from palliative care [42, 43].

We have shown a high proportion of lung cancer patients who die within 30 days of diagnosis are older, have comorbid disease and are admitted through the emergency department. These characteristics, available at diagnosis, may be useful prognostic factors to guide decisions on early assessment for palliative care for lung cancer patients. Further research is needed on the palliative care needs of elderly patients admitted through the emergency department with suspected lung cancer.

Patients who die shortly after diagnosis are more likely to die in hospital so reporting place of death by survival time may be useful to evaluate interventions to reduce deaths in acute hospitals. It would also highlight sub groups of patients who might benefit from early assessment for and referral to palliative care.

## Abbreviations

AJCC: American joint committee on cancer
CCI: Charlson comorbidity index
CSO: Central statistics office
ESRI: Economic and social research institute
HIPE: Hospital in-patient enquiry
ICD: International classification of diseases
ICD-O3: International classification of disease for oncology, 3rd edition
IQR: Interquartile range
NCRI: National Cancer Registry Ireland
